# Reconstruction of engineered yeast factory for high yield production of ginsenosides Rg3 and Rd

**DOI:** 10.3389/fmicb.2023.1191102

**Published:** 2023-06-19

**Authors:** Yuan Lin, Yi Na Wang, Guang Hui Zhang, Geng Chen, Qing Hui Yang, Bing Hao, Sheng Chao Yang

**Affiliations:** ^1^State Key Laboratory of Conservation and Utilization of Bio-Resources in Yunnan, The Key Laboratory of Medicinal Plant Biology of Yunnan Province, National and Local Joint Engineering Research Center on Germplasms Innovation and Utilization of Chinese Medicinal Materials in Southwest China, Yunnan Agricultural University, Kunming, Yunnan, China; ^2^Key Laboratory of Medicinal Plant Biology, Yunnan Agricultural University, Kunming, Yunnan, China; ^3^College of Agronomy and Biotechnology, Yunnan Agricultural University, Kunming, Yunnan, China; ^4^Yunnan Characteristic Plant Extraction Laboratory, Kunming, Yunnan, China

**Keywords:** *Panax notoginseng*, ginsenosides, glycosyltransferase, biosynthesis, engineered yeast

## Abstract

*Panax notoginseng* is one of the most valuable traditional Chinese herbs. The main active ingredients, dammarane-type ginsenosides, show multiple pharmacological activities. Recently, the key UDP-dependent glycosyltransferases (UGTs) involved in the biosynthesis of common ginsenosides have been widely studied. However, only a few UGTs that catalyze ginsenoside formation have been reported. This study further investigated the new catalytic function of 10 characterized *UGTs* from the public database. *PnUGT31*(*PnUGT94B2*) and *PnUGT53* (*PnUGT71B8*)exhibited promiscuous sugar-donor specificity of UDP-glucose and UDP-xylose, which could catalyze the glycosylation of C20-OH sites and elongation of the sugar chain at the C3 and/or C20 sites. We further analyzed the expression patterns in *P. notoginseng* and predicted the catalytic mechanisms of *PnUGT31* and *PnUGT53* using molecular docking simulations. Moreover, different gene modules were built to increase the yield of ginsenosides in engineered yeast. The metabolic flow of the proginsenediol (PPD) synthetic pathway was enhanced by LPPDS gene modules based on the engineered strain. The resulting yeast was constructed to produce 1.72 g/L PPD in a shaking flask, but cell growth was significantly inhibited. EGH and LKG gene modules were constructed to achieve high-level production of dammarane-type ginsenosides. The production of G-Rg3 controlled by LKG modules increased 3.84 times (254.07 mg/ L), whereas the G-Rd titer reached 56.68 mg/L after 96 h in shaking flask culture under the control of all modules, both of which yielded the highest values for known microbes.

## Introduction

The underground part of *Panax notoginseng* has been widely used for treating cancer and cardiovascular and cerebrovascular diseases (Zhang et al., 2018). Damarane-type saponin is considered to be the main active agent responsible for various pharmacological activities. At present, more than 200 ginsenosides have been found in *P. notoginseng*, and dammarane-type saponins account for 98%. More specifically, dammarane-type ginsenoside Rb1 (G-Rb1), Rb2 (G-Rb2), Rg1 (G-Rg1), Re (G-Re), Rc (G-Rc) and notoginoside R1 (N-R1) account for more than 90% of the total saponins (Wan et al., 2006; Wei et al., 2018). Depending on the position of the hydroxyl group, dammarane-type saponins can be divided into proginsendiol-type (PPD-type) and proginsentriol-type (PPT-type) saponins. More than 20 dammarane-type saponins have been tested in animal experiments and preclinical studies, and the proginsenodiol-type ginsenosides, G-CK, G-Rh2, G-F2, and G-Rg3, have shown significant anticarcinogenic activity (Chen et al., 2013; Wang et al., 2018; Chen et al., 2019; Liu et al., 2020). It is worth noting that G-Rg3 has entered the stage of clinical studies as an efficient antitumor agent (Sun et al., 2017).

Dammarane-type ginsenosides are tetracyclic triterpenes. The hydroxyl groups at the C3, C6, and C20 sites of the skeleton can be glycosylated by different sugar donors to form ginsenosides. During the biosynthesis of ginsenosides, the final step of glycosylation is normally catalyzed by UDP-dependent glycosyltransferase (UGT) with the supplementation of UDP-sugar donors such as UDP-glucose (UDP-Glu), UDP-xylose (UDP-Xyl), UDP-rhamnose, and UDP-arabinose, and substituting sites can form sugar chains that combine multiple sugar groups. Glycosyltransferases are a group of multi-gene superfamilies which can transfer sugar from the substrate to specific receptor molecules. All UGTs involved in ginsenoside biosynthesis belong to family 1 glycosyltransferases (GT1 family), which uses 5 ‘-uridine diphosphate - sugar as glycosyl donors. Based on the similarity of amino acid sequences, *UGTs* from plants can be divided into 94 subfamilies and clades that have not yet been classified (Ross et al., 2001). Previous reports have shown that the *UGTs* involved in the biosynthesis of dammarane-type saponins are mainly distributed in the UGT71, UGT74, and UGT94 subfamilies of plants.

Currently, the acquisition of dammarane-type saponins depends heavily on the raw plant materials, and their content is easily affected by various environmental factors. In fact, more than 200 types of dammarane-type ginsenosides only account for approximately 2% of the dry weight of 3-year-old *P. notoginseng*, that is hard to meet the market demand for rare ginsenosides. Synthetic biology offers a new strategy for producing valuable compounds via heterologous organisms. To overcome this yield limit, more than 10 *UGT* genes have been engineered in yeast to produce ginsenosides. Although some achievements have been made in the microbial production of dammarane-type ginsenosides, the highest yield of proginsenodiol-type ginsenosides, such as Rg3 is still less than 100 mg/L (Jiang et al., 2022).

To date, five versions of the genome and abundant transcriptomes of *P. notoginseng* have been published, and nearly 100 *UGTs* have been identified in *P. notoginseng* (Wang et al., 2020; Yang et al., 2021). These rich data from the genome, transcriptome, and metabolome provide a solid foundation for exploring the functions of UGTs. Here, based on the *UGTs* obtained from previous studies, we further identified two UDP-xylose-dependent glycosyltransferases capable of biosynthesizing vinaginsenosides R16 and R18, as well as UDP-Glu for glycosylation at the C20 site and sugar chain extension. Based on the reconstructed PPD-producing *Saccharomyces cerevisiae*, we used these two *UGTs* to establish a cell factory to produce part of the PPD-type ginsenosides as an alternative source for expanding the industrial production of ginsenosides to protect wild populations of *Panax* plants, such as *P. notoginseng*.

## Materials and methods

### Strains and materials

*Saccharomyces cerevisiae* strain BY4742 (Wuhan Miaoling Biotechnology Co., LTD, China) was used as the initial strain for engineering. *Escherichia coli* strain BL21 (DE3; TransGen Biotech, China) and *S. cerevisiae* strain W303-1B were used for heterologous expression. *E. coli* DH5α and Trans-T1 chemically competent cells (TransGen Biotech) were used for the cloning of UGTs. The codon-optimized gene was synthesized by Tsingke Biotechnology (Beijing, China). All primers and strains used in this study are listed in Supplementary Tables S1, S2. The standards and substrate compounds PPD, PPT, and ginsenosides (>98%) were purchased from DeSiTe Biotechnology (Chengdu, China) and PuRuiFa Technology (Chengdu, China).

### Cloning and heterologous expression of recombinant *Pn*UGT proteins

The coding sequence of UDP-glycosyltransferase was determined by PCR-amplified from *P. notoginseng* and cloned into the pEASY vector (TransGen Biotech, Beijing, China). Briefly, *UGT* genes were ligated into the pET28a vector with a 6 × His-tag using the Golden Gate Assembly Kit (NEB, USA) and transformed into *E. coli* BL21 (DE3). *UGT* genes with a C-terminal 6 × His-tag were ligated into the YCplac22 vector using the ClonExpress II One Step Cloning Kit (Vazyme Biotech Co., Ltd., Nanjing, China).

After the sequence was correctly constructed into *E. coli*, positive clones were selected and cultured in 4 mL LB medium containing 100 mg/L kanamycin (Kan) at 37°C for 12–16 h, and the bacteria solution was transferred into 400 mL LB + Kan^+^ medium for further culture until the OD_600_ value reached 0.5–0.8. The medium was induced with 0.1 mM isopropyl-β-D-thiogalacto-pyranoside (IPTG) at 15°C for 16 h. The cells were collected and resuspended in 100 mM lysis buffer (pH 7.4). The cell suspension was lysed at 4°C and 2000 bar with a high pressure cell fragmentation instrument (Constant Systems, Britain). The supernatant obtained by centrifugation was concentrated and used for enzymatic reactions.

The recombinant yeast strains were cultivated in 2 mL YPD medium at 30°C for 16–18 h, and then transferred to 50 mL YPD medium for further culture for 2 days. The cells were collected and suspended in 15 mL of PBS (pH 7.4). The yeast cells were broken at 4°C and high pressure of 3,000 bar. The supernatant obtained by centrifugation was concentrated and used for enzymatic reactions.

### Enzyme assays

Enzymatic assays of UGTs were performed in random combinations, consisting of UDP-glucose, UDP-arabinose, UDP-rhamnose, and UDP-xylose as sugar donors with 11 ginsenoside substrates. The 100 μL reaction system included 100 mM phosphate buffer (pH = 7.4), 2 mM UDP-sugar donors, 1 mM ginsenoside, 50 μL crude UGT enzyme, incubated at 35°C for 12 h and terminated by adding 100 μL of methanol. The lysates of *E. coli* harboring the pET28a vector and *S. cerevisiae* harboring the YCplac22 vector were used as negative controls. After centrifugation at 14,000 ×*g* for 10 min, the supernatant was filtered through a 0.22 mm nylon syringe filter and prepared for HPLC and LC–MS analysis. After the enzymatic assays system was expanded ten times, the reactants were incubated in a thermostatic oscillator with a revolution of 800 rpm/min, and n-butanol was used at a ratio of 1:1 to terminate the reaction. The supernatant obtained after centrifugation was subjected to NMR analysis.

### Real-time reverse transcriptase polymerase chain reaction quantification

Omega Plant RNA Kit (Omega Bio-Tek, China) was used to isolate the total RNA of *P. notoginseng*, and the HiScript II One Step RT-PCR Kit (Vazyme, China) was used to obtain cDNA. *PnUGT53* and *Pn3-29* were amplified by real-time PCR (RT-qPCR) using SYBR Green Realtime PCR Master Mix (Vazyme, China). The primers are listed in Supplementary Table S1.

### Construction, cultivation, and metabolite extraction of yeast strains

The establishment of the gene expression cassette required two rounds of PCR cloning. Q5 High-Fidelity DNA Polymerase Cloning Kit (NEB, United States) was used to amplify basic fragments containing promoters, genes, terminators, selective markers, and homologous fragments of the yeast genome in the first round. Each basic fragment has a special homologous sequence of 40–75 bp on either side for recombination or fusion PCR. In the second round, gene expression cassettes were obtained by fusion of basic fragments. All fusion fragments were purified, quantified, and co-transformed into yeast cells using the standard lithium acetate method for assembly and integration. Correct clones were first inoculated into 2 mL synthetic dropout medium and grown at 30°C until the OD_600_ value reached 0.8–1.0, and the bacteria solution was subsequently transferred into 30 mL YPD medium and grown at 30°C for 5–6 days. To extract ginsenosides from the fermentation culture cells, the cells were collected and suspended in 6 mL methanol. After ultrasonication for 30 min, the separated supernatants were used for HPLC analysis.

### Chemical analysis

The HPLC analysis was performed using an Agilent 1200 series preparative HPLC system (Santa Clara, CA, United States). Chromatographic separation of ginsenosides from enzyme assay products was carried out at 25°C on a Phenomenex 00D-4,627-E0 Kinetex 5 μm Biphenyl 100 Å, LC Column (100 mm × 4.6 mm, 5 μm, United States). The detection wavelength for the ginsenosides was 203 nm. The gradient elution system comprised water (A) and acetonitrile (B). There are two gradient programs for separation, the first gradient program: 0 ~ 4 min (15–30% B), 9 ~ 15 min (40–42% B), 18 ~ 25 min (60–100% B); the second gradient program: 0 ~ 6 min (20–30% B), 8 ~ 12 min (40–42% B), 25 ~ 30 min (90–100% B), and the flow rate was kept at 1.0 mL/min.

LC–MS and LC–MS/MS was performed using a Micro ToF MS instrument (Bruker Daltonics) equipped with an Agilent HP1100 series LC system. The chromatographic column and gradient elution programs are the same as in HPLC analysis. The products were identified in negative mode. All spectra were recorded in negative ion mode over 50–1,000 m/z under 6.01 L/min dry gas flow, 203 nm detection wavelength, 180°C dry temperature, 1 bar nebulizer pressure, and 14.5 kV probe voltage.

To isolate of new glycosylated products from enzyme assays, the glycosylated products were collected from the n-butanol phase of the enzyme assay suspension and dried using a Termovap Sample Concentrator to yield a dry residue. The dry residue was dissolved in methanol and further purified with an Agilent Agilent ZORBAX SB-C18 column (9.4 × 250 mm 5 μm, 2.2 μm, Shimadzu, CA, United States) (solvent A: water; solvent B: acetonitrile; the gradient: 0 ~ 7 min (40–50% B), 16 ~ 20 min (60–70%); flow rate 2 mL/min). The resulting methanol phase was collected and completely evaporated to yield the crystallized product, which was weighed and dissolved in deuterium pyridine-d5 for NMR analysis. ^1^H and ^13^C NMR spectra were obtained using an 800 MHz Bruker Avance III spectrometer.

For the separation of ginsenosides from yeast fermentation products, an Agilent Poroshell 120 EC-C18 column (100 mm × 3.0 mm, 2.7 μm) was used for gradient elution at 35°C. The gradient elution system comprised water (A) and acetonitrile (B). There are two gradient programs for separation, the first gradient program: 0 ~ 6 min (15–30% B), 11 ~ 17 min (40–42% B), 25 ~ 27 min (100–100% B); the second gradient program: 0 ~ 7 min (30–40% B), 7 ~ 12 min (40–42% B), 13 ~ 19 min (60–90% B), 21 ~ 25 min (100–100% B), and the flow rate was kept at 0.8 mL/min. The calibration curves of the standard samples were generated based on the integrated peak of HPLC, which was used to quantify of G-CK, G-Rg3, G-Rh2, G-Rd and PPD.

## Results

### Functional characterization of *PnUGT31* from *Panax notoginseng*

**Figure 1:**
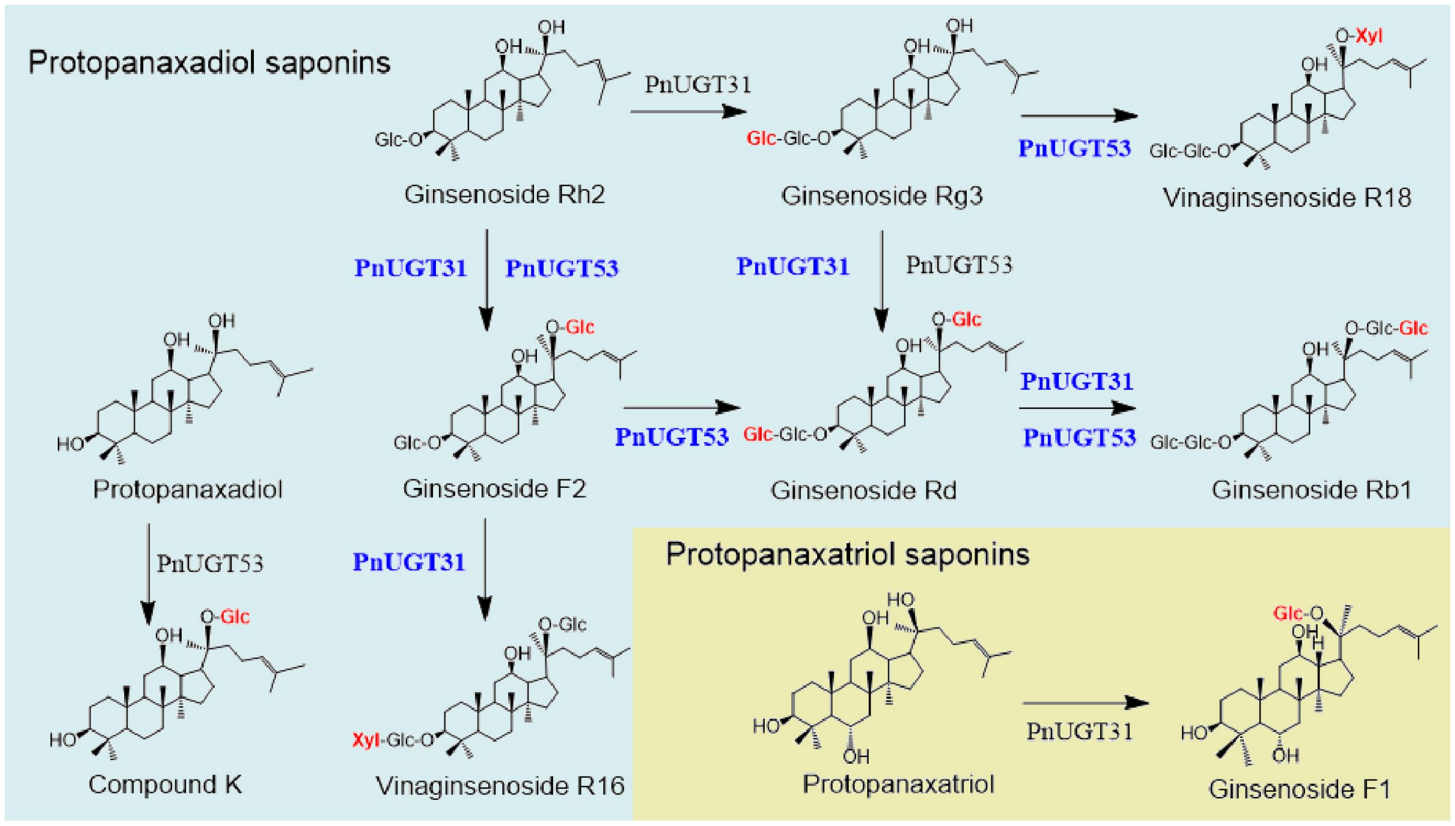
Role of glycosyltransferase (*Pn*UGT31 and *Pn*UGT53) from *P. notoginseng* in ginsenosides biosynthesis pathway.

**Figure 2:**
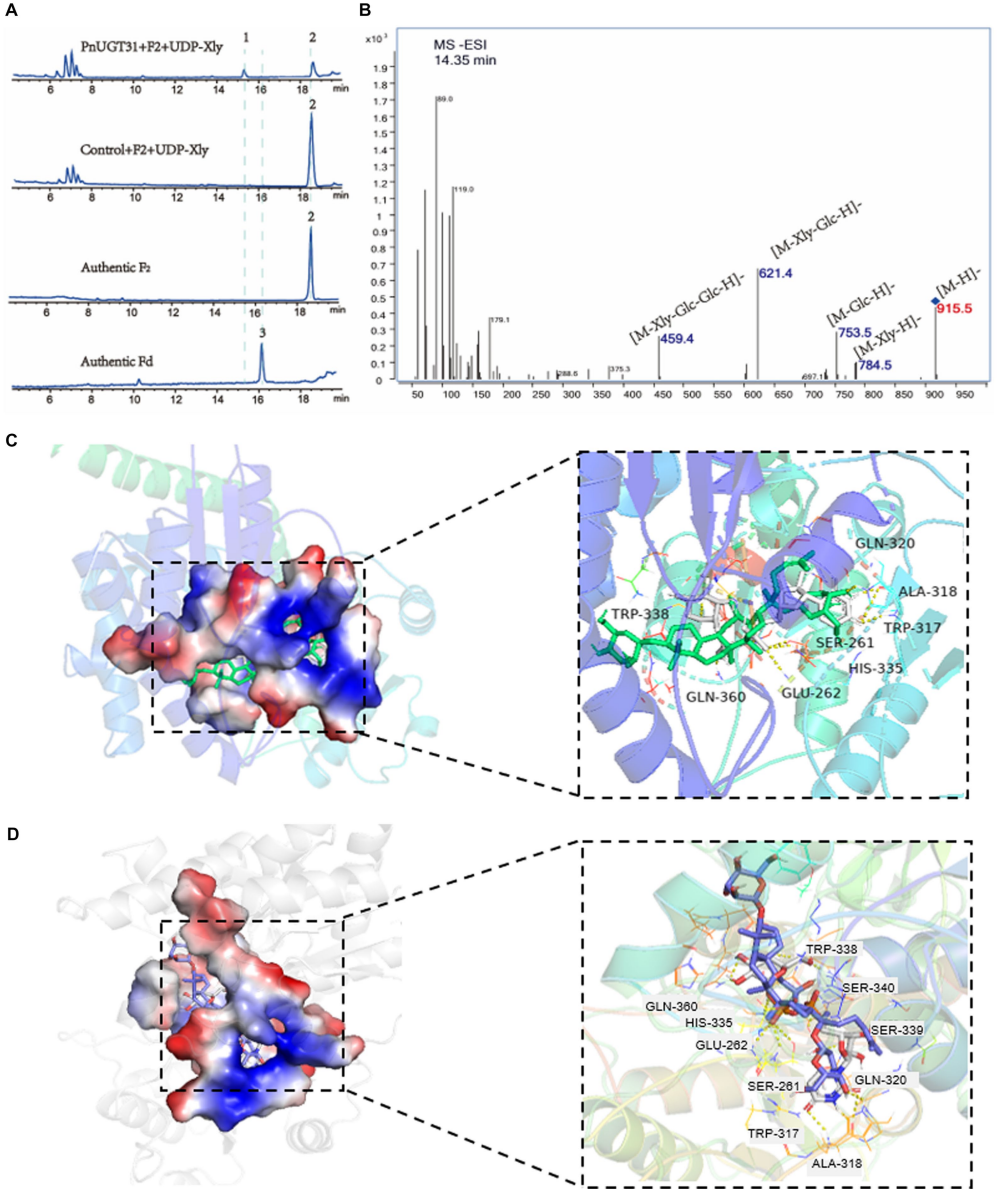
Function and enzymatic reaction mechanism study of *Pn*UGT31. The blue fonts represent the first reported functions in this study. **(A)** HPLC results of enzyme assays including PnUGT31, G-F2 and UDP-xylose, peak 1 presents new products 1, peak 2 presents G-F2, peak 3 presents G-Fd. **(B)** MS/MS results of product 1. **(C,D)** shows a simulation of molecular docking between protein *Pn*UGT31 with ligands. The ligands in **(C)** are UDP-xylose and G-F2; The ligands in **(D)** are UDP-glucose and G-F2.

A total of 116 *UGT*s from *P. notoginseng* were obtained from the PanaxGDB database^1^ (Lin et al., 2022). According to previous studies, UGTs that catalyze dammarane-type ginsenoside belong mainly to the UGT 74, 94, and 71 families. Therefore, we screened 19 candidate *UGTs* from these families as candidate genes which closely related to other UGTs that catalyze dammarane-type ginsenosides in the phylogenetic tree (Supplementary Figure S1). Information regarding these 19 candidate *UGT* genes is provided in Supplementary Table S3. We found that *UGT*s involved in the biosynthesis of dammarane-type ginsenosides were mainly highly expressed in the rhizomes or roots of *P. notoginseng* (Supplementary Figure S2). Finally, ten candidate *UGT*s with high-level expression in roots or rhizomes were selected from these 19 *UGTs* for functional verification (Supplementary Figure S2). The UDP-xylose-dependent catalytic activity of the 10 *PnUGTs* was screened by heterologous expression of recombinant *Pn*UGT proteins in *E. coli* or *S. cerevisiae*. Eleven dammarane-type ginsenosides were selected as substrates for enzyme assays. HPLC and LC-MC results showed that *Pn*UGT31 (*PnUGT94B2*) could catalyze xylosylation of G-F2 to produce an unknown product 1 with a fragment feature of 915.5 (m/z, [M-H]^−^) mass (Figure 1). NMR analysis revealed that product 1 was vinaginsenoside R16 (V-R16). The results showed *Pn*UGT31 catalyzes the elongation of the second sugar chain at the C20 site of G-F2 with xylose to produce V-R16 (Figures 1, 2A,B; Supplementary Figures S3A–C).

The sequence characteristics of *Pn*UGT31 were consistent with those previously reported *Pn*3-31, which catalyzes G-Rh2 to produce G-Rg3 (Wang et al., 2020). In our study, *Pn*UGT31 showed catalytic activity to produce G-Rg3, G-F2, G-Rd, V-R16,G-Rb1 and G-F1 starting from G-Rh2 with UDP-glycose and UDP-xylose as the substrates (Figure 1; Supplementary Figure S4). Moreover, *Pn*UGT31 showed the capability of glycosylating the C20-OH and elongating the sugar chain at the C3 or/and C20 sites. These new catalytic functions of *Pn*UGT31 have not been reported yet. When the molar mass ratio of the sugar donor to the substrate is 5:1, *Pn*UGT31 can catalyze the formation of G-F2 and G-Rg3 from G-Rh2 in an enzyme system simultaneously (Supplementary Figure S4). Although parts of G-Rg3 were consumed by continuous glycosylation, the peak area of G-Rg3 was still higher than that of G-F2, indicating that *Pn*UGT31 preferred elongation of the sugar chain at the C3 and/or C20 sites over glycosylation of the C20-OH site (Supplementary Figure S4). Interestingly, we found that *Pn*UGT31 could not catalyze C20-OH glycosylation in the presence of an extra group at the C6-position of the saponin skeleton.

The three-dimensional protein structure of *Pn*UGT31 was predicted and molecular blind docking prediction was conducted with 11 PPD-type ginsenosides and four UDP-sugar donors. There were seven UDP-xylose and 11 UDP-glucose hydrogen-bonding residues between these two sugar-donor ligands and *Pn*UGT31, mainly distributed in the Ser261-Glu262, Trp317-Gln320, His335-Trp338, and Asp359-Gln360 regions (Figures 2C,D). The key residues responsible for the hydrogen-bonding activity between *Pn*UGT31 and the four dammarane-type ginsenosides (G-Rh2, G-Rg3, G-F2, and G-Rd) were Trp317-Gln320, Gln228-Arg229, and His20-Ser22 (Supplementary Figure S5). Trp317-Gln320 are the conserved residues in the PSPG region, where is the overlapping region that sugar donor ligands interact with the protein (Supplementary Figure S5).

### Functional characterization of *PnUGT53* from *Panax notoginseng*

**Figure 3:**
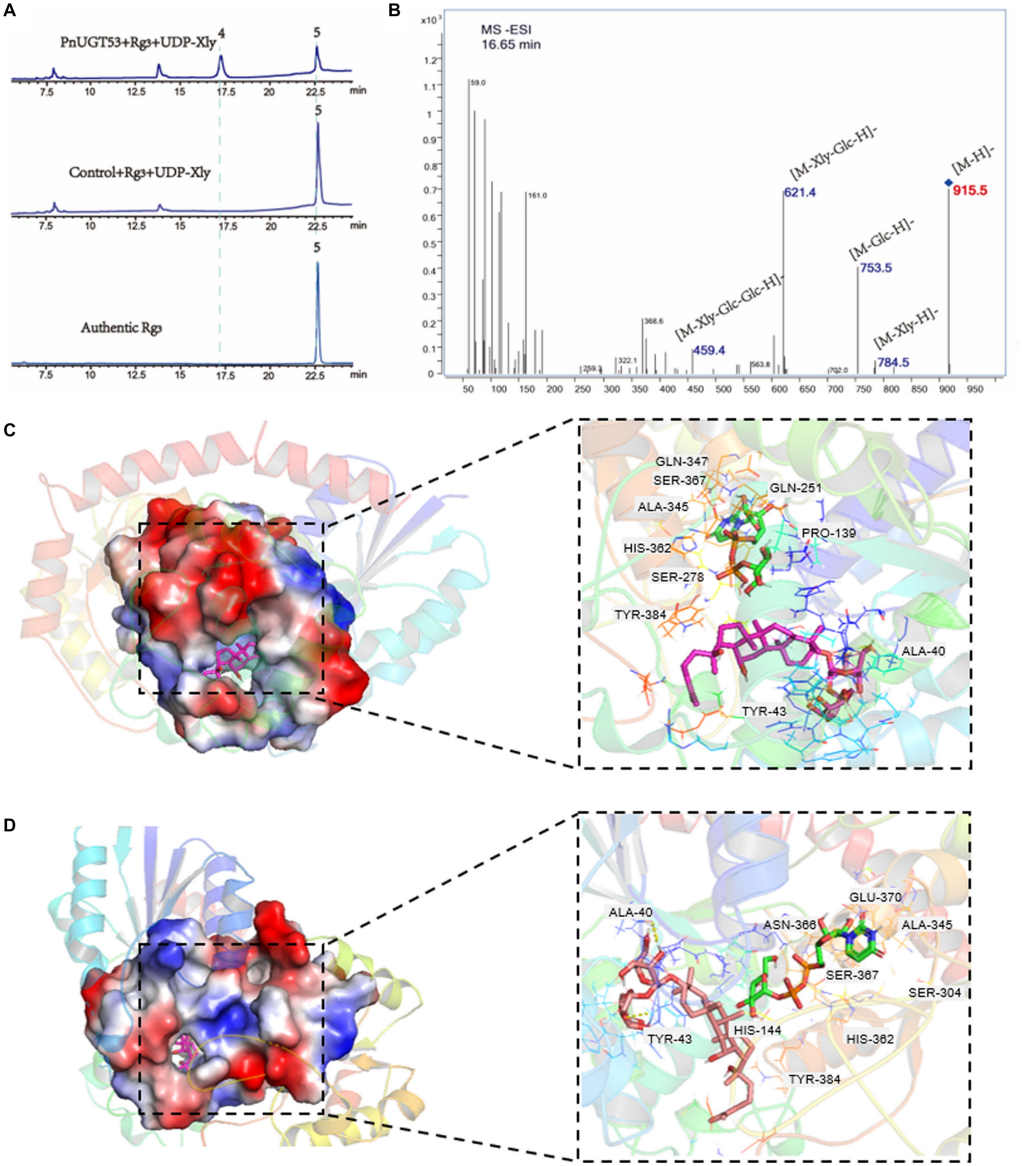
Function and enzymatic reaction mechanism of *Pn*UGT53. **(A)** HPLC results of enzyme assays including *Pn*UGT53, G-Rg3 and UDP-xylose, peak 4 was new products 2, peak 2 was G-Rg3. **(B)** MS/MS results of new product 2. **(C,D)** Shows a simulation of molecular docking between protein *Pn*UGT53 with ligands. The ligands in **(C)** are UDP-xylose and G-Rg3; The ligands in **(D)** are UDP-glucose and G-Rg3.

*In vitro* enzyme assays, we found that *Pn*UGT53 (*Pn*UGT71B8) could catalyze the xylosylation of G-Rg3 to form the known product 2 with 915.5 (m/z, [M-H]-) mass, which was identified as vinaginsenoside R18 (V-18) by NMR analysis (Supplementary Figures S3D–F; Figures 3A,B). This is the first demonstration of a key UGT enzyme in the biosynthesis of V-18. *Pn*UGT53 could catalyze the C20-OH site of PPD, PPT, and G-Rg3 to produce G-CK, G-F1, and G-Rd with the supplementation of UDP-glucose, respectively (Supplementary Figure S4; Figure 1). It also catalyzed the continuous glycosylation of G-Rh2 via the G-F2 and G-Rd pathways to generate G-Rb1 (Supplementary Figure S4; Figure 1). *Pn*UGT53 catalyzed the glycosylation of C20-OH and elongation of the sugar chain at the C3 and/or C20 sites but favored the glycosylation of the C20-OH site under these two conditions. G-Rg3, the product of the extension of the sugar chain, was not found, even though the ratio of sugar donors was higher *in vitro* assays (Supplementary Figure S4; Figure 1). After glycosylation at C6-OH, *Pn*UGT53 could not further catalyze the glycosylation of C20-OH in the saponin skeleton.

*PnUGT53* is highly homologous to the previously reported *Pn3-29* (99.79%). *Pn*3-29 can catalyze G-Rg3, PPD, and PPT to generate G-Rd, G-CK, and G-F1, respectively. However, the catalytic capacity of *Pn*UGT53 differs from that of the reported *Pn*3-29, the sequences of these two genes were compared. The results showed that there were only differences in one amino acid and three nucleobases. Alphafold2 predicted that the spatial folding structure of the two proteins was completely consistent, and the different amino acids were not located in the key region of the substrate-bonding site (Supplementary Figure S6). Eleven ginsenosides and four UDP-sugar donors were selected as ligands for molecular docking (Supplementary Figure S6; Figures 3C,D). The results showed that their differential loci were not key sites for substrate selection. Therefore, it remains to be seen whether these two genes have similar catalytic functions. Molecular blind docking prediction showed two to six hydrogen bonds were observed between different receptor sites and *Pn*UGT53 in the Ala40-Asp45 and Arg81-Ile84 regions (Figures 3C,D; Supplementary Figure S6). *Pn*UGT53 forms eight and seven hydrogen bonds with UDP-xylose and UDP-glucose ligands, respectively, and these two sugar ligands exhibit a hydrogen-bonding reaction with residues Ala345, Ser367, Typ384, and His362 (Figures 3C,D; Supplementary Figure S6). These four residues may be active sites for UDP sugar binding.

**Figure 4:**
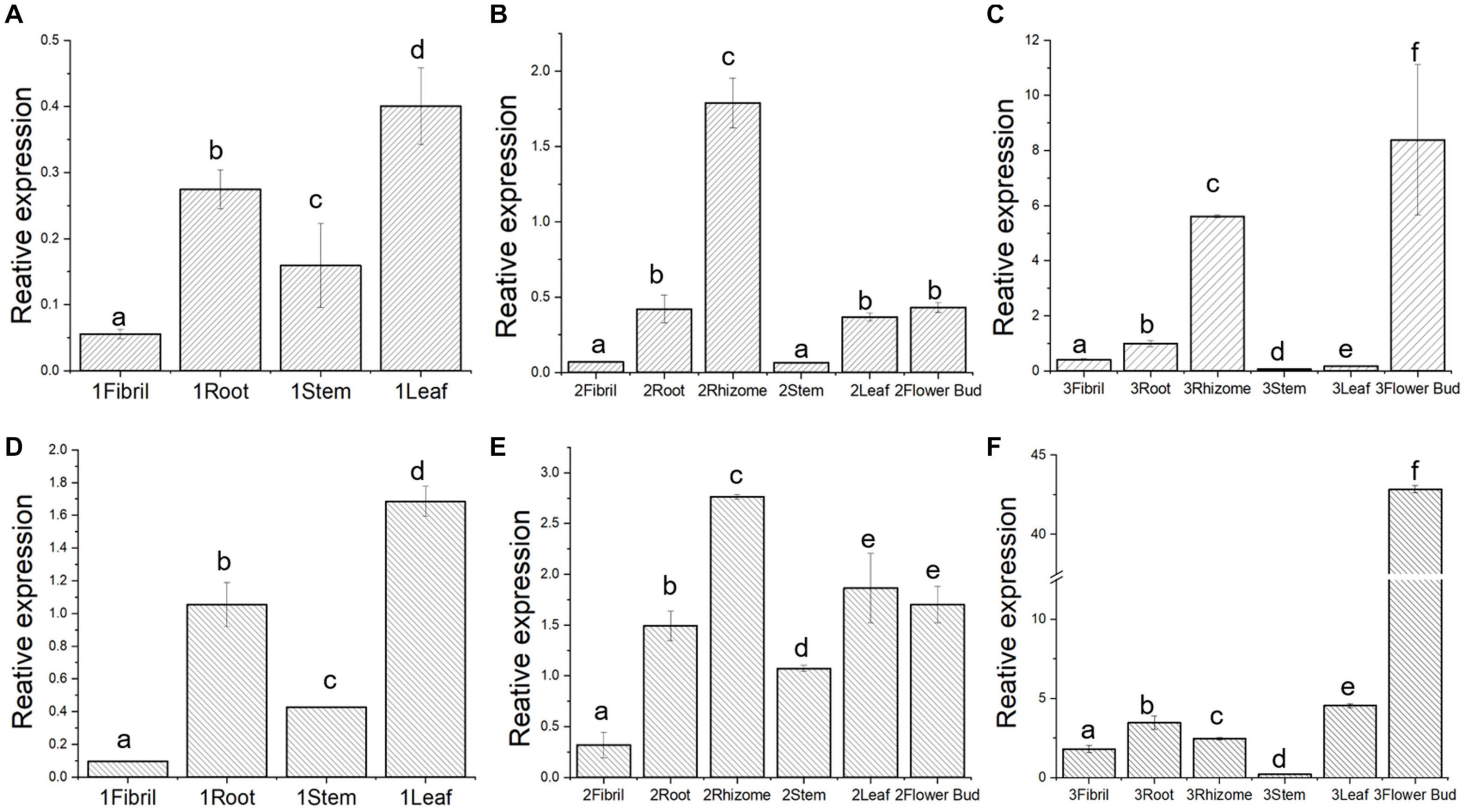
Expression pattern and enzymatic reaction mechanism study of *Pn*UGT53 and *Pn*3-29. **(A–C)** Relative expression of *Pn*UGT53 in different tissues of 1-, 2- and 3-year-old *P. notoginseng*; **(D–F)** Relative expression of *Pn*3-29 in different tissues of 1-, 2- and 3-year-old *P. notoginseng*. *p* < 0.05, different letters represent significant differences between different parts.

To further verify whether *Pn3-29* and *PnUGT53* have the same expression pattern in *P. notoginseng*, we designed primers for their differential nucleobases for RT-qPCR. The gene expression trends of *Pn3-29* and *PnUGT53* in the different tissues of 1-year-old and 2-year-old *P. notoginseng* plants were similar (Figures 4A,B,D,E). Interestingly, The expression levels of *PnUGT53* and *Pn3-29* significantly differented in the 3-year-old *P. notoginseng*. The relative expression level of *PnUGT53* has significantly increased in flower buds and rhizomes of 3-years old *P. notoginseng*, whereas the relative expression level of *Pn3-29* in 3-years old *P. notoginseng* was significantly increased in the flower buds (Figures 4C,F). Moreover, the relative expression levels of *Pn3-29* in different tissues of 1–3 years old plants were higher than those of *PnUGT53* (Figure 4). Single SNPs within the gene may directly affect the expression levels of *PnUGT53* and *Pn3-29* in *P. notoginseng*.

### Design and construction of engineered yeast for PPD production

**Figure 5:**
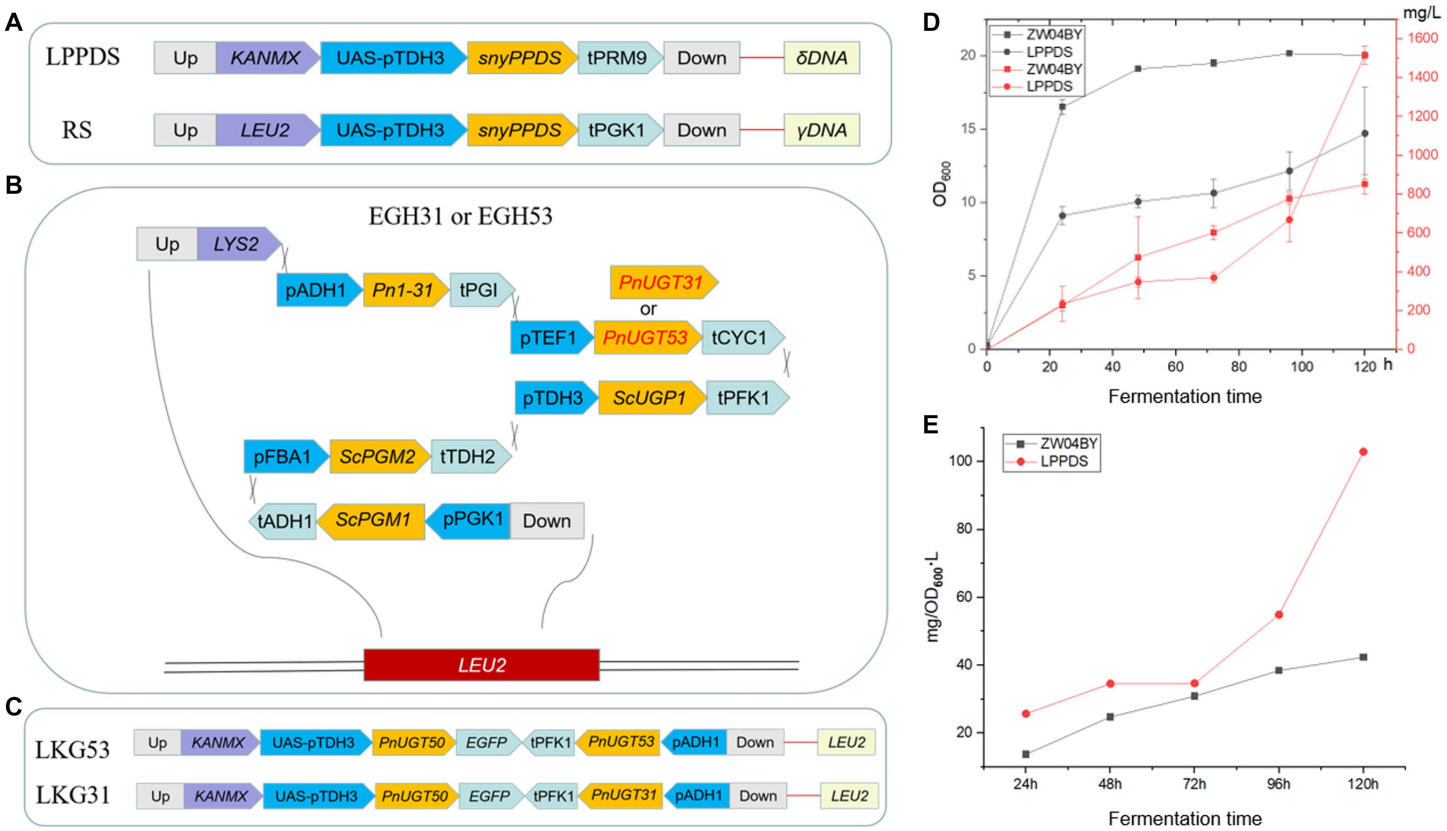
Schematic representation of the gene modular construction and PPD yield of strain LPPDS. **(A)** Construction of LPPDS and RS module. **(B)** Construction of EGH31 and EGH53 module. **(C)** Construction of LKG31 and LKG53 module. **(D)** The yield of PPD produced by strain LPPDS and ZW04BY. **(E)** Ratio of PPD yield to OD_600_ of strain LPPDS and ZW04BY.

PPD is a direct precursor of G-Rh2. The yeast strain BY4742 was employed as the starting strain according to a previous study to construct a PPD-producing yeast strain ZW04BY (Wang et al., 2019). Strain ZW04BY produced 652 mg/L and 851 mg/L PPD after 96 h and 120 h in a shake flasks (Figure 5D; Supplementary Figure S7). Our results show that the PPD yield of strain ZW03BY in a shake flasks was higher than in a previous study (529.0 mg/L). There was still a possibility of converting more accumulated dammarenediol (DM) into PPD. To increase the metabolic flow of PPD biosynthesis, codon-optimized *PgPPDS* controlled by the *TDH3 + UAS_TEF1-CIT1-CLB2_* promoter was inserted into the strain ZW04BY. Previous studies have shown that the selection of combinatorial promoters enhances gene expression (Blazeck et al., 2012). To increase the copy number of the *PgPPDS* in yeast, the *δ* sequence was selected as inserted locus, while G418 (geneticin) was selected as resistance selection tags (Parekh et al., 1996; Wang et al., 1996). This engineered strain was constructed based on LPPDS gene modules named LPPDS (Figure 5A). Compared with the control strain ZW04BY, the PPD yield of strain LPPDS reached 1.72 g/L, double the control yield (Figure 5D; Table 1). Although the engineered strain LPPDS could show significantly improved PPD production, the OD_600_ value showed that the cell growth of the strain LPPDS was significantly inhibited compared to the control, which might be due to the metabolic inhibition (Figure 5E).

**Table 1:** The yield of ginsenosides produced by engineering yeast.

	Yield (mg/L)				
Strains	PPD	G-CK	G-Rh2	G-Rg3	G-Rd
ZW04BY	851 ± 51.87	–	–	–	–
LPPDS	1722.10 ± 80.72	–	–	–	–
EGH31	947.32 ± 78.95	–	–	52.48 ± 21.02	–
EGH53	1027.66 ± 60.22	20.48 ± 0.20	4.40 ± 0.13	–	–
LKG31	721.36 ± 71.37	–	12.80 ± 9.59	254.06 ± 56.49	–
LKG53	745.75 ± 147.29	–	35.42 ± 5.37	–	–
LKG31RS	795.41 ± 83.54	–	0.72 ± 0.14	164.58 ± 24.54	26.21 ± 12.86
LKG53RS	908.46 ± 64.58	–	54.57 ± 6.36	–	–
LKG53EGHLPPDS	1313.75 ± 87.33	–	55.70 ± 1.44	–	56.68 ± 16.21

### High-level production of proginsenodiol-type ginsenosides

Both *Pn*UGT31 and *Pn*UGT53 catalyze continuous glycosylation of G-Rh2 to G-Rb1. In this study, *PnUGT31* and *PnUGT53* were placed under the control of the TEF1 promoter and CYC1 terminator and inserted into the *EGH1* (*Yir007w*) locus of *S. cerevisiae* strain ZW04BY. The effect of glucosidase on the desugarization and degradation of ginsenosides can be reduced by knocking out EGH1, which can hydrolyze various *β*-glucosides (Dai et al., 2014; Zhuang et al., 2017; Figure 5B; Supplementary Table S2). The fragment contained the *Pn*1-31 gene was inserted to increase the production of G-Rh2 precursor in yeast (Supplementary Figure S2). To increase the UDP-glucose stock, phosphoglucomutase 1 (*Sc*PGM1), phosphoglucomutase 2 (*Sc*PGM2), and UDP-glucose-phosphorylase (*Sc*UGP1) genes were controlled by the *PGK1*, *FBA1*, and *TDH3* promoters and *ADH1*, *TDH2* and *PFK1* terminators, respectively (Figure 5B; Supplementary Table S2). All the above genes constituted the EGH-31 and EGH-53 gene modules, and the corresponding engineered strains were named EGH31 or EGH53, respectively (Figure 5B; Supplementary Table S2).

**Figure 6:**
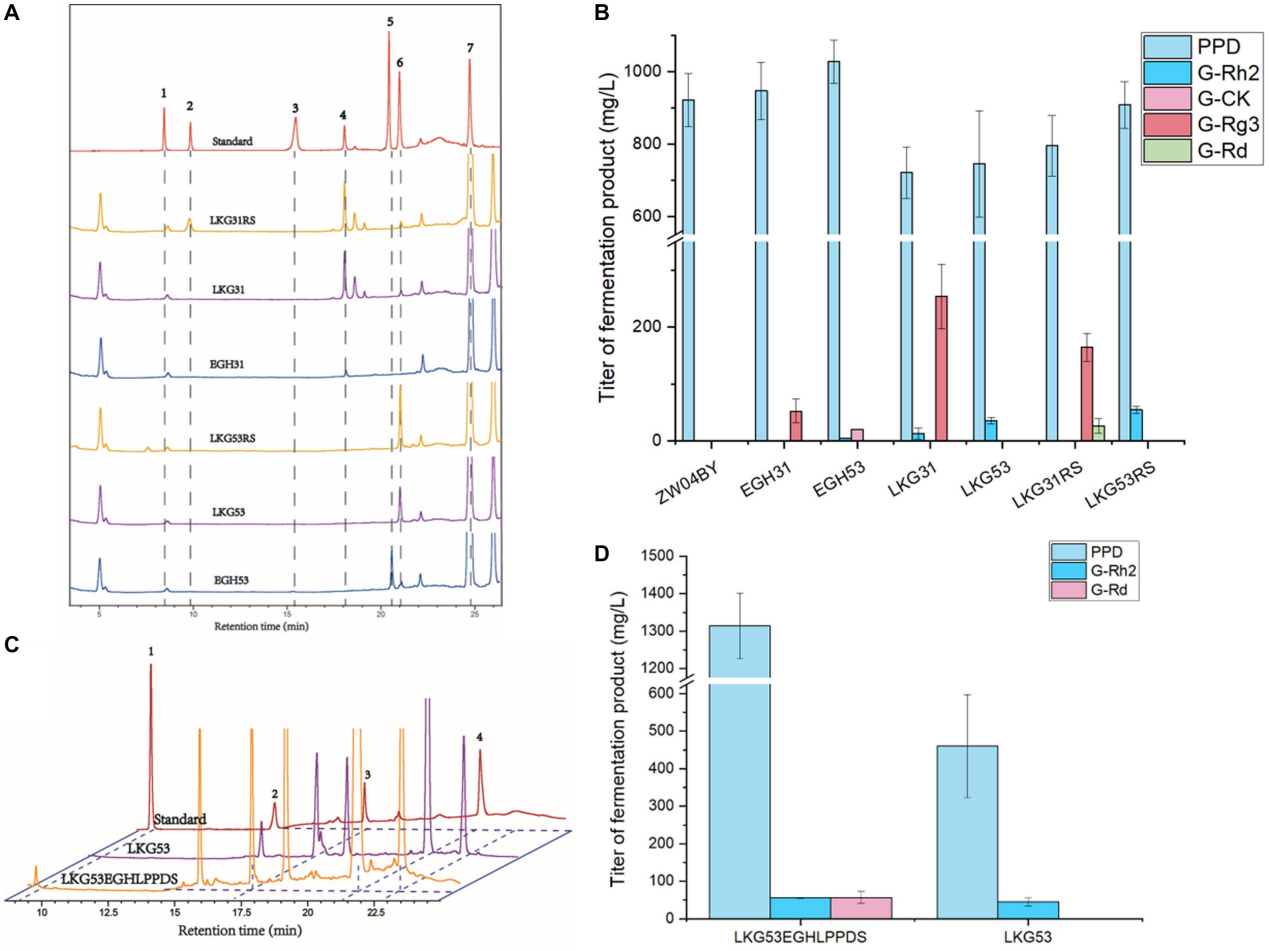
Ginsenoside products from engineered yeast. **(A)** HPLC results of engineered yeast after 120 h in shaking flask. The gradient elution system 1 for separation of ginsenosides from yeast products. Peak 1 represents ginsenoside Rb1; Peak 2 represents ginsenoside Rd.; Peak 3 represents ginsenoside F2; Peak 4 represents ginsenoside Rg3; Peak 5 represents ginsenoside CK; Peak 6 represents ginsenoside Rh2; Peak 7 represents PPD. **(B)** The yield of ginsenosides produced by engineered yeast after 120 h in shaking flask. **(C)** HPLC results of ginsenoside Rd. produced by engineered strain LKG53EGHLPPDS. Peak 1 represents ginsenoside Rd., peak 2 represents ginsenoside Rg3, peak 3 represents ginsenoside Rh2 and peak 4 represents PPD. The gradient elution system 2 for separation of ginsenosides from yeast products. **(D)** The yield of ginsenosides produced by strain LKG53EGHLPPDS in shaking flask after 96 h.

The HPLC results of the yeast fermentation extracts showed that strain EGH31 produced peaks of DM, PPD, and G-Rg3, but intermediate G-Rh2, suggesting that G-Rh2 was completely consumed in the biosynthesis of saponins in the downstream pathway (Figures 6A,B). There was also evidence of G-Rg3 formation, but no G-F2 peak was observed, which further confirmed that *Pn*UGT31 preferentially catalyzes sugar chain extension (Figure 6A). The products of strain EGH53 showed peaks for DM, PPD, G-Rh2, and G-CK, and the titer of G-CK was significantly higher than that of G-Rh2, further verifying that *Pn*UGT53 prefers the glycosylation of C20-OH rather than catalyzing the extension of the sugar chain (Figure 6A). No peaks for G-Rd and G-Rb1 were detected in the fermentation extracts of strains EGH31 and EGH53, indicating that *Pn*UGT53 and *Pn*UGT31 have weak catalytic synthesis abilities for G-Rd and G-Rb1 or generate fewer precursor substances (Figure 6B). The results showed strain EGH31 could produce 0.95 ± 0.08 g/L PPD and 52.48 ± 21.02 mg/L G-Rg3, whereas strain EGH53 could produce 1.03 ± 0.06 g/L PPD, 20.48 ± 0.20 mg/L G-CK and 4.40 ± 0.13 mg/L G-Rh2 (Figure 6B; Table 1).

### Accumulation of key precursors

The precursor G-Rh2 produced by the engineered yeasts EGH31 and EGH53 was consumed, resulting in low production of ginsenosides in the downstream pathway. Insufficient precursor G-Rh2 did not generate sufficient G-Rb1. To improve the titer of G-Rh2, SnyPnUGT50, the expression controlled by the *TDH3 + UAS_TEF1-CIT1-CLB2_* promoter, was selected to catalyze PPD to generate G-Rh2. Sny*Pn*UGT50 significantly increased the titer of G-Rh2 from PPD in yeast (Wang et al., 2019). The insertion of *PnUGT53* and *PnUGT31* fragments promoted ginsenosides biosynthesis in the downstream pathway. *LEU2* was selected as the insertion locus to recover leucine selection labels. The gene modules mentioned above were named LKG-31 or LKG-53, and the corresponding engineered strains were designated LKG31 and LKG53, respectively (Figure 5C; Supplementary Table S2). After shaking for 120 h, strain LKG53 consumed more PPD than EGH53. The absence of G-CK indicated that UGT53 was consumed to catalyze PPD to produce G-Rh2, which increased 8-fold the titer of G-Rh2, from 4.40 ± 0.13 mg/ L to 35.42 ± 5.37 mg/ L. In addition, the G-Rg3 yield of strain LKG31 (254.07 ± 56.49 mg/ L) was 3.84 times higher than that of strain EGH31 (52.48 ± 21.02 mg/L; Figure 6B; Table 1). These results show that the LKG module significantly increased the titer of G-Rg3 in *S. cerevisiae*.

To increase the PPD precursor yield, two methods were employed to increase the conversion rate of DM to PPD. The insertion of the LPPDS module at the *δ* sequence site may inhibit of cell growth. To exclude cell growth inhibition caused by the insertion site, the engineered yeast strains LKG31RS or LKG53RS were constructed based on the LKG31 and LKG53 strains by replacing with *γ* sequence as the insertion site. The engineered yeast strains LKG31RS and LKG53RS, and their control strain LKG, were cultured in a shake flask for 120 h. These results revealed the presence of G-Rd in the strain LKG31RS (Figure 6A). Meanwhile, the PPD titers of strains LKG31RS and LKG53RS increased by 10.27 and 21.76%, respectively. Compared to the control group, the total saponin production in the downstream pathway also increased (Figure 6B; Table 1). In addition, the OD_600_ of these strains was not significantly difference compared to the control strain LKG31 or LKG53 (data not shown). HPLC results showed that the peak area of DM decreased by 80.03%, whereas the total saponin production increased by 23.3%. The peak area of DM from strain LPPDS decreased by 85.02% compared to the control strain ZW04BY, and total saponin (PPD) production increased by 132.3%. Therefore, the introducting of the RS module alleviated cell-growth inhibition to a certain extent and increased the PPD conversion rate, which was slightly lower than that of the LPPDS module. Quantitative analysis showed that strain LKG31RS produced 795.41 ± 83.54 mg/L PPD, 0.72 ± 0.14 mg/L G-Rh2, 164.58 ± 24.54 mg/L G-Rg3 and 26.21 ± 12.86 mg/L G-Rd. Strain LKG53RS produced 908.46 ± 64.58 mg/L PPD and 54.57 ± 6.36 mg/L G-Rh2 (Figure 6B; Table 1).

Owing to the restriction of the enzyme activity of *Pn*UGT31 and *Pn*UGT53, appropriately increasing the accumulation of the precursors PPD and G-Rh2 is helps improve the biosynthesis of downstream saponins. The resulting strain was named LKG53EGHLPPDS after stacking the above three modules. The G-Rh2 titer of strain LKG53EGHLPPDS (55.67 ± 1.44 mg/L) increased by 23.86% compared with that of strain LKG53 (44.97 ± 12.01 mg/L) following incubation in a shake flask for 96 h (Figures 6C,D; Table 1). The PPD yield of strain LKG53EGHLPPDS was 1313.75 ± 87.33 mg/L, and that of strain LKG53 was 460.15 ± 137.48 mg/L, Which is a 185% increase (Figures 6C,D; Table 1). Strain LKG53EGHLPPDS also produced 56.68 ± 16.21 mg/L G-Rd, twice higher than that of strain LKG31RS (Figures 6C,D; Table 1).

## Discussion

*P. notoginseng* has the highest agricultural output value in traditional Chinese medicine, owing to various bioactive ginsenosides. The biosynthetic pathways and heterologous biosynthesis of ginsenosides and saponins have recently attracted significant attention. In this study, known *UGTs* from previous studies on ginsenoside biosynthesis were screened from the PDB (panaxGDB) to discover new catalytic functions of *Pn*UGT53 and *Pn*UGT31. Moreover, the key UGT (*Pn*UGT53) of V-18 biosynthesis was identified for the first time.

A previous study reported that UGTs involved in dammarane-type ginsenoside biosynthesis might catalyze different substrates even with high sequence homology (98.11–99.37%; Zhao et al., 2020). We found that the sequence homology between *PnUGT53* and *PnGT95* was 98.74%. *Pn*GT95 was reported to catalyze the glycosylation of hydroxyl groups at C20 and C6 sites of dammarane-type saponins (Yu et al., 2019). However, *Pn*UGT53 catalyzed the glycosylation of C20-OH, extended the sugar chain of dammarane-type saponins, and accepted UDP-xylose as a sugar donor in our study. *Pn*UGT53 and *Pn*UGT95 exhibited similar but different catalytic functions. Their expression patterns in different tissues of 3-year-old *P. notoginseng* plants were also distinct. *Pn*UGT53 was highly expressed in the flowers, whereas *Pn*UGT95 was highly expressed in the rhizomes of 3-year-old *P. notoginseng*. Other studies showed that more than 40 UGTs catalyze ginsenosides in the UGT94 family of *P. ginseng*, and the amino acid identity between *UGTs* is higher than 98%, but the catalytic activities or functions are quite different (Yang et al., 2020).

To further investigate the catalytic mechanisms of *Pn*UGT31 and *Pn*UGT53, we performed molecular docking simulations. Previous studies have shown that ten conserved residues in the PSPG region are directly linked to sugar donors (Li et al., 2007). Correspondingly, in the predicted model, *Pn*UGT53 and *Pn*UGT31 had four and six amino acid residues connected to sugar ligands, respectively, which also corresponded to the conserved residues in the PSPG region. Among the 44 amino acids in the PSPG region, tryptophan, aspartic acid/glutamic acid, and glutamine at positions 22, 43, and 44 are easily hydrogen-bonded to sugar ligands (Osmani et al., 2009). However, *Pn*UGT31 and *Pn*UGT53 lacked the corresponding hydrogen bonds at positions 43 and 44. The overlap of the donor and acceptor pockets in *Pn*UGT31 may increase the probability of an interaction between the two. When a protein performs a catalytic function, its structure is not invariable, and interdomain interactions between the C-terminal- and N-terminal domains are often found (Li et al., 2007). Protein structure prediction and molecular docking technology can be used to predict the interactions between proteins and ligand molecules, but their accuracy still needs to be verified by further experiments.

To increase the production of ginsenosides, the engineered ZW04BY was reconstructed to produce two times higher PPD than the reported yield. This may be due to differences in the number of gene copies obtained by yeast transformation in single cells, which causes biological differences among strains, or the selection of G418-resistant transformants. In this study, G418 resistance screening was performed twice for the LPPDS and LKG modules of strain LKG53EGHLPPDS. A higher concentration of G418 (400 mg/L) was selected for the second screening, which was applied to strain LKG53 as a chassis cell. After replacing strain LKG31 as the chassis cell, transformants could not be obtained through G418 resistance screening, which the copy number and expression level of the *KANMX* gene in strains LKG53 and LKG31 might have caused. In addition, the insertion of the LPPDS module resulted in the inhibition of the cell growth of the strain LPPDS and strain LKG53EGHLPPDS. We hypothesized that because 2, 3-oxsqualene is a common precursor of the synthesis of steroidal saponins and PPD, the production of PPD was greatly increased and the precursor 2,3-oxsqualene was consumed in large quantities, which might restrict the biosynthesis of steroidal saponins necessary for cell growth in plastids eventually causing cell growth inhibition (Parks and Casey, 1995). It could also be that δ sequences are involved in the reverse transcription process in yeast cells, and excessive consumption δ sequences may impose a metabolic burden on cells. Therefore, we designed the RS module to change the insertion site into γ sequences. The results showed that cell growth inhibition was alleviated, but the transformation efficiency of DM was lower than that of the LPPDS module.

Both *Pn*UGT31 and *Pn*UGT53 continuously catalyzed G-Rh2 to produce G-Rb1. However, *PnUGT31* and *PnUGT53* were constructed into engineered yeast, and G-Rb1 was not detected in the fermentation products of all engineered yeasts. The same situation was observed in the engineered yeast platforms *Pn*3-31 and *Pn*3-29 in a previous study (Wang et al., 2020). We suspect that this may be due to the lower accumulation of the G-Rd precursor or the weak activity of the enzyme that catalyzes G-Rd to produce Rb1. Moreover, results showed that codon optimized *PnUGT31* in strains was able to produce more downstream ginsenosides than *PnUGT53*. Enzymatic activity is the main limiting factor in the production of PPD-type saponins by engineered yeast. However, the adaptability of genes to the yeast expression systems must also be considered. The gene expression level of *SynCYP2* from *P. notoginseng,* which catalyzes PPD to generate protopanaxatriol (PPT), was lower than that from *P. ginseng* in engineered yeast, even though the abundance of PPT-type ginsenosides in *P. notoginseng* was higher than that in *P. ginseng* (Li et al., 2021).

In this study, engineered yeasts producing PPD, G-Rh2, G-Rg3, G-Rd, and other ginsenosides were successfully constructed, but there is still much work to be done to improve the production of dammarane-type ginsenosides by *S. cerevisiae.* The previously reported *Pn*UGT33 was selected as the key enzyme for G-Rg3 production to construct engineered yeast. Yeasts overexpressing *ScPGM1*, *ScPGM2*, and *ScUGP1* genes produced 51 mg/L G-Rg3 in the optimized YPD medium, which was lower than that of LKG31 (254.07 mg/ L). In this study, the EGH module was not successfully constructed into the LKG31RS genome for unknown reasons, and we failed to increase the G-Rd titer of strain LKG53EGHLPPDS (56.68 mg/L), yet it was the highest yield from a known engineered yeast.

## Conclusion

In this study, new catalytic activity of PnUGT31 and PnUGT53 was identified and utilized in yeast cell factory producing damarane-type ginsenosides using metabolic engineering. We constructed appropriate gene modules, and finally increased the titer of G-Rg3 and G-Rd in yeast. This study established an efficient method for the production of G-Rg3 and G-Rd. Meanwhile, the synthetic biology strategy of this study can also be used for heterologous synthesis of other natural products in yeast.

## Data availability statement

The datasets presented in this study can be found in online repositories. The names of the repository/repositories and accession number(s) can be found in the article/Supplementary material.

## Author contributions

BH, YL, and SY conceived the study. YL, GC, and YW performed the experiments. GZ, YL, and SY the designed experiments. YL and QY analyzed the data. BH and YL drafted the manuscript. YL, BH, and SY reviewed and edited the manuscript. All authors contributed to the article and approved the submitted version.

## Funding

This work was supported by Major Science and Technology Projects in Yunnan Province (2019ZF011-1), Fundamental Research Project of Yunnan (202101AS070037), Science and Technology Innovation team of Yunnan (202105AE160011), The Major Science and Technique Programs in Yunnan Province (202102AE090042), Yunnan Characteristic Plant Extraction Laboratory (2022YKZY001), the First Projects of Science and Technology Plan in the Biomedical field in 2021 (202102AA310048), and National Natural Science Foundation of China (grant nos. 81960691 and 82160727).

## Conflict of interest

The authors declare that the research was conducted in the absence of any commercial or financial relationships that could be construed as a potential conflict of interest.

## Publisher’s note

All claims expressed in this article are solely those of the authors and do not necessarily represent those of their affiliated organizations, or those of the publisher, the editors and the reviewers. Any product that may be evaluated in this article, or claim that may be made by its manufacturer, is not guaranteed or endorsed by the publisher.

## Footnotes

^1^ http://panaxGDB.ynau.edu.cn

## Abbreviations

qRT-PCR: real-time reverse transcriptase polymerase chain reaction
PPD: protopanaxadriol
PPT: proginsentriol-type
UGT: UDP-dependent glycosyltransferase
HPLC: high performance liquid chromatography
LC–MS: liquid chromatography-mass spectrometry
NMR: nuclear magnetic resonance spectroscopy
LC–MS/MS: chromatography-tandem mass spectrometry
G-: ginsenside
V-: vinaginsenosides
GT: glycosyltransferases
UDP-Glu: UDP-glucose
UDP-Xyl: UDP-xylose
