# Supplementary material for: Reconstruction of engineered yeast factory for high yield production of ginsenosides Rg3 and Rd
Source: Front Microbiol. 2023 Jun 19;14:1191102. doi: 10.3389/fmicb.2023.1191102 (PMC10315489; doi:10.3389/fmicb.2023.1191102)

Fig S1. Phylogenetic tree of 116 *PnUGTs* and *UGTs* that involved in saponins biosynthesis. The blue shaded part is the 19 candidate UGTs: Pno06G008659.t1, Pno06G008735.t1, Pno10G002218.t1, Pno05G004370.t1, Pno11G015482.t1, Pno08G004291.t1, Pno08G004292.t1, Pno11G000939.t1, Pno11G000940.t1, Pno11G000942.t1, Pno05G004358.t1, Pno05G000496.t1, Pno01G004255.t1, Pno08G004993.t1, Pno11G000829.t1, Pno10G001943.t1, Pno06G008612.t1, Pno05G000501.t1 and Pno11G000824.t1. Information regarding these 19 *UGT*s is provided in panaxGDB database (http://panaxGDB.ynau.edu.cn).

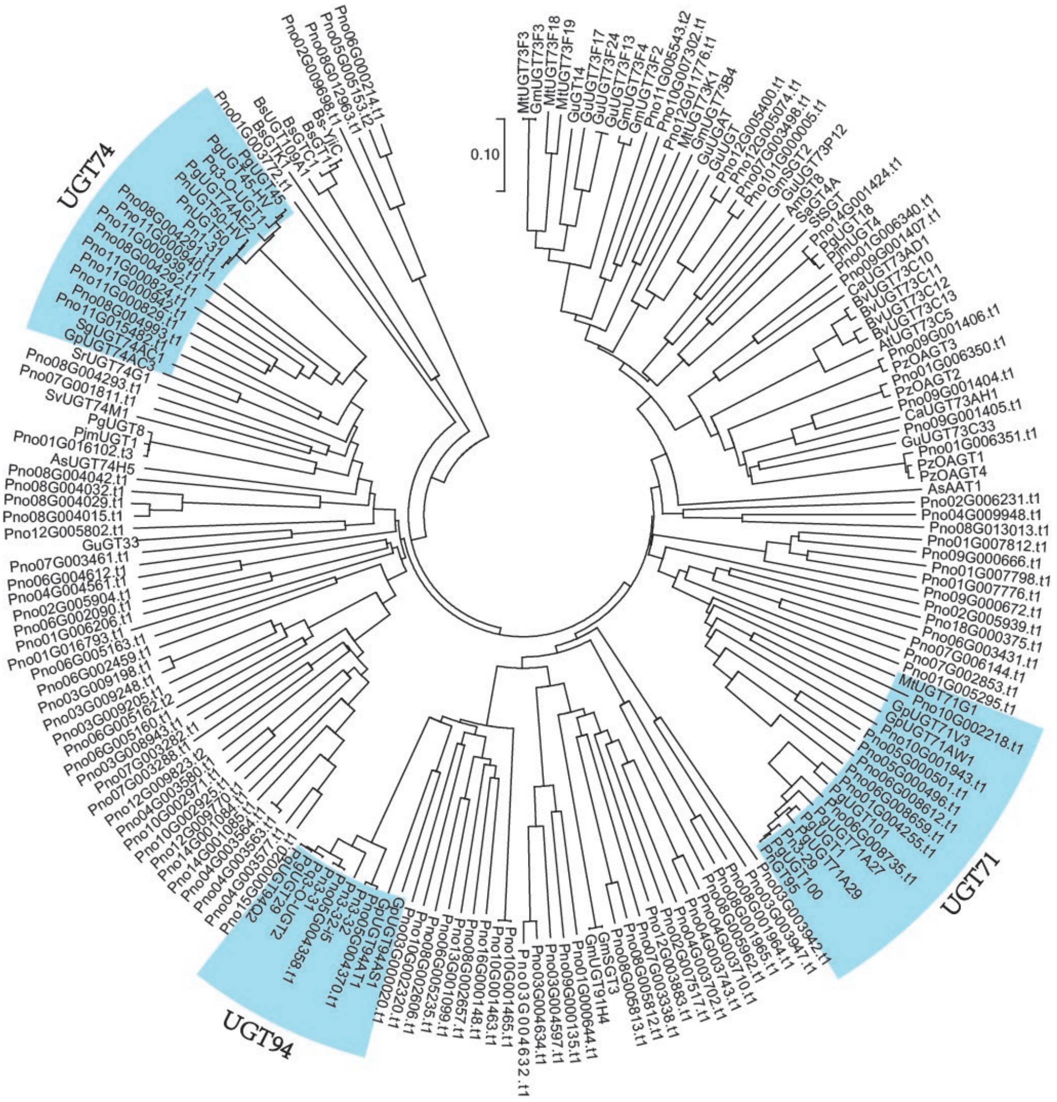

Fig S2. The expression profile of *UGT*s in *P. notoginseng*. The red words is the published *UGT*s, and the blue words is the candidate *UGTs* that has not been functionally characterized. All the genes represented by coloured fonts are expressed at high-levels in roots and rhizomes. De nove transcriptome assembly data from http://panaxGDB.ynau.edu.cn.

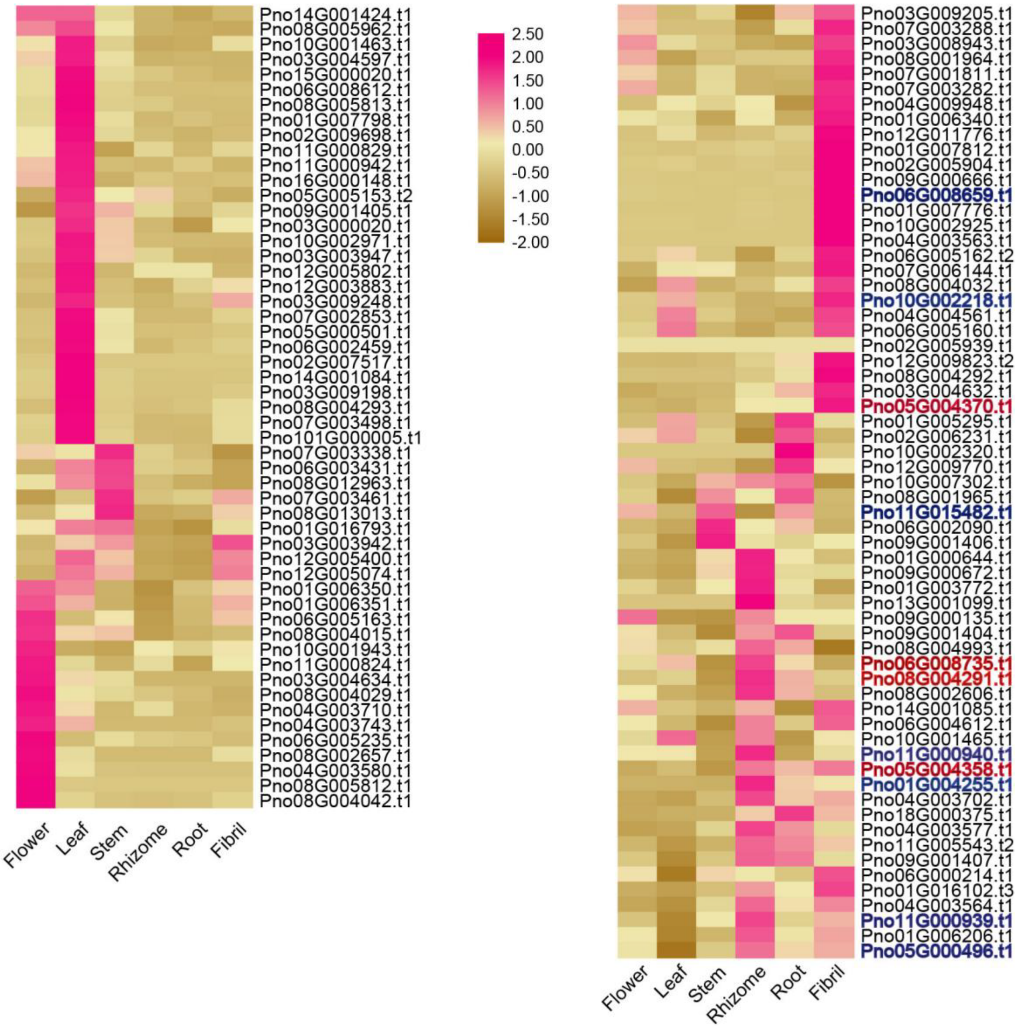

Fig S3. The ^{13}C-NMR and ^{1}H-NMR results of new products from enzyme assays. (A). The ^{13}C-NMR results of new product 1. (B). The ^{1}H-NMR results of new product 2. (C). The molecular structure of new product 1. (D). The ^{13}C-NMR results of new product 2. (E). The ^{1}H-NMR results of new product 2. (F). The molecular structure of new product 2.

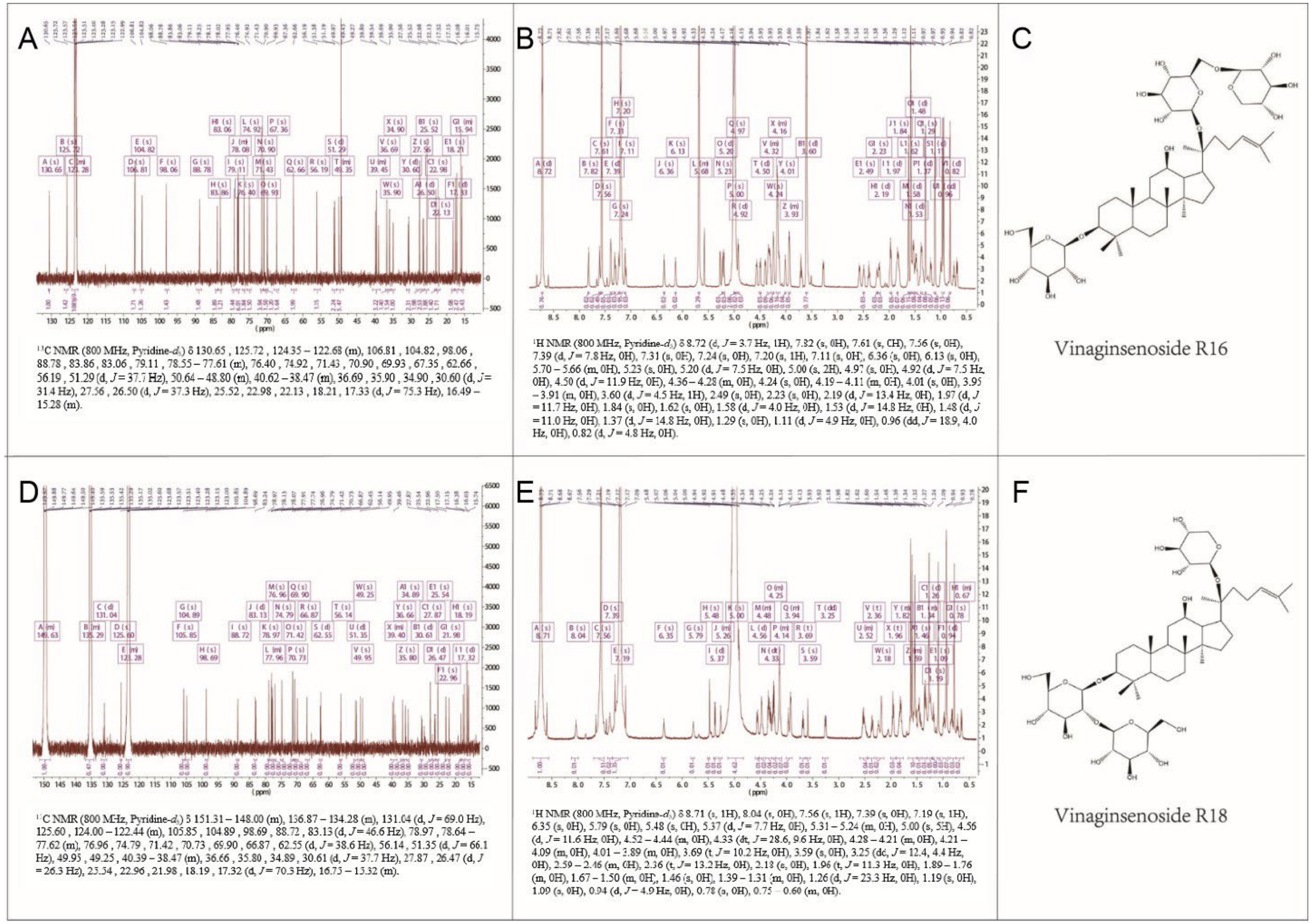

Fig S4. HPLC analysis of glycosylation activity of glycosyltransferase (*Pn*UGT31 and *Pn*UGT53) to dammarane-type saponins. (A) Catalytic activity of *Pn*UGT31 on proginsendiol-type saponins. (B), (C) and (D) The catalytic activity of *Pn*UGT53 on proginsendiol-type saponins; (E) Catalytic activity of *Pn*UGT53 on proginsentriol-type saponins. (A) and (C) use the gradient elution system 1 for chromatographic separation of ginsenosides from enzyme assays, (B), (D) and (E) use the gradient elution system 2.

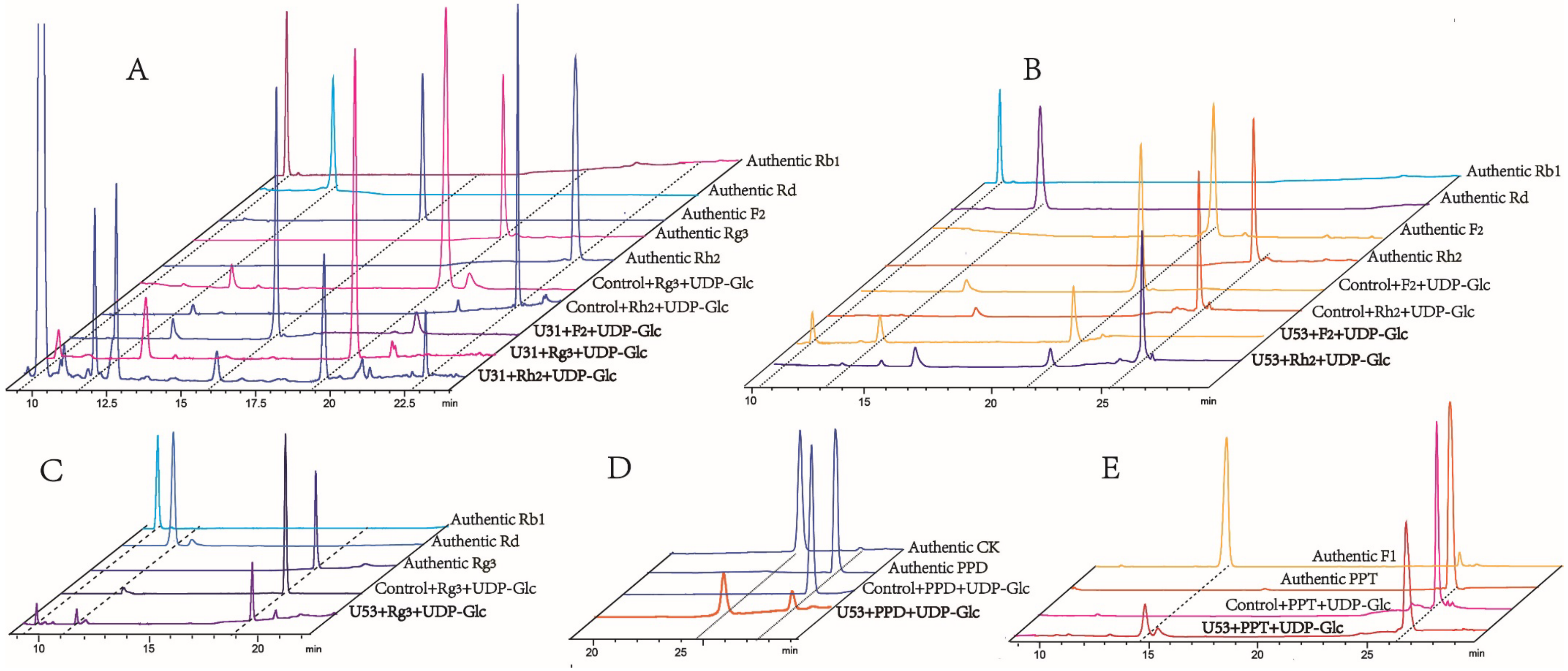

Fig S5. Molecular docking prediction of *Pn*UGT31 and *Pn*UGT53 with different ginsenosides. Fig (A)-(E) shows the protein structure of *Pn*UGT53. Fig (F)-(I) shows the protein structure of *Pn*UGT31. All the sugar ligands in the picture are UDP-glucose. (A) and (F). the ligand was ginsenoside Rh2; (B) and (G). the ligand was ginsenoside Rg3; (C) and (H).the ligand was ginsenoside F2; (D) and (I). the ligand was ginsenoside Rd; (E). the ligand was PPD.

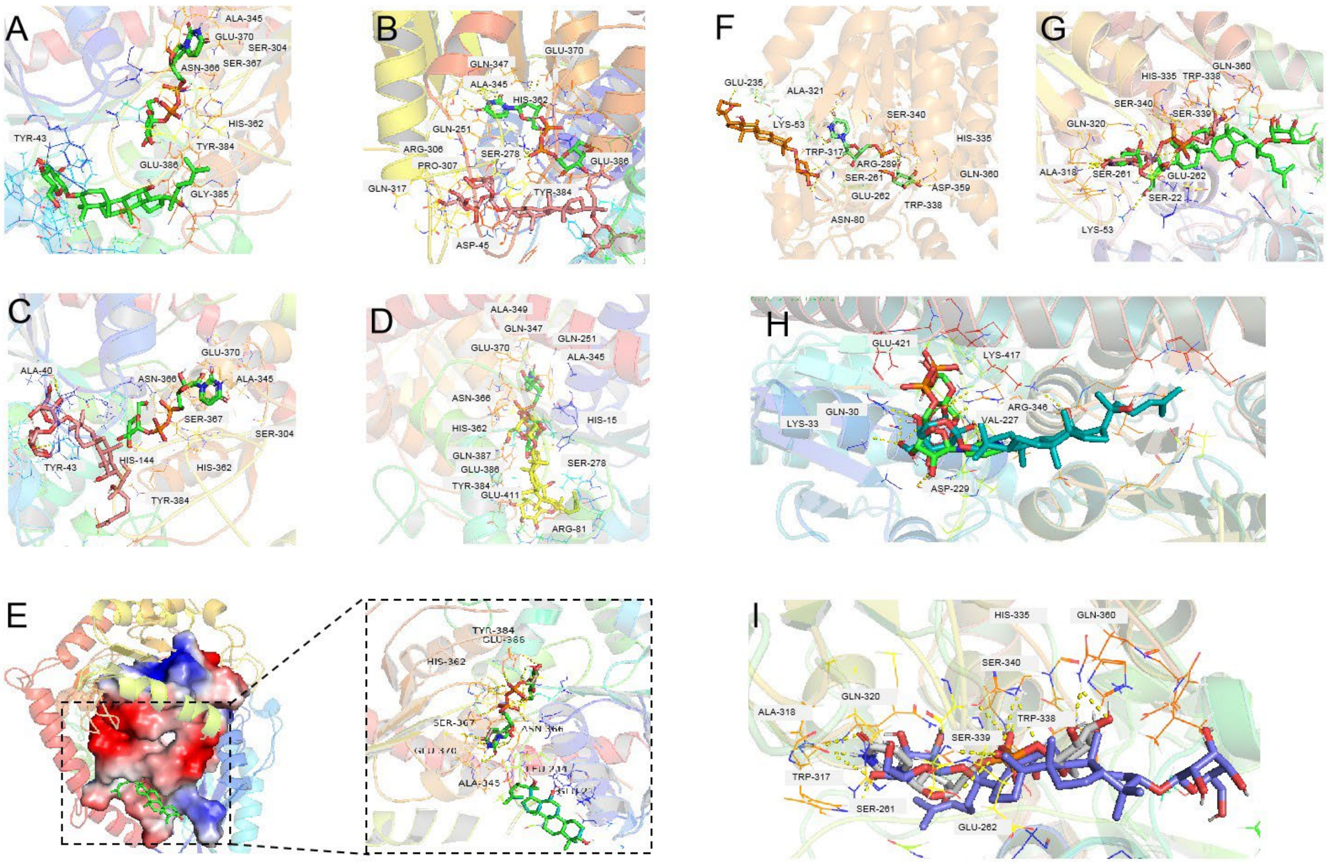

Fig S6. Location of difference amino acid residues between *Pn*UGT53 and *Pn*3-29 on the structure of *Pn*UGT53 protein. The sugar ligand is in fushcia, the substrate molecular ligand is in yellow, the differential amino acidresidue (Val/*Pn*UGT53) is in red.

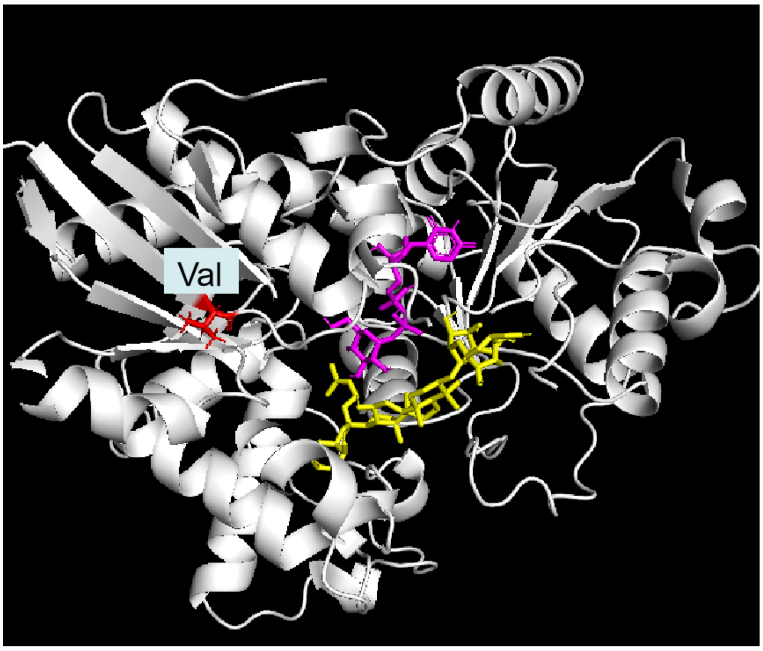

Fig S7. HPLC analysis of fermentation products from strains ZW04BY.

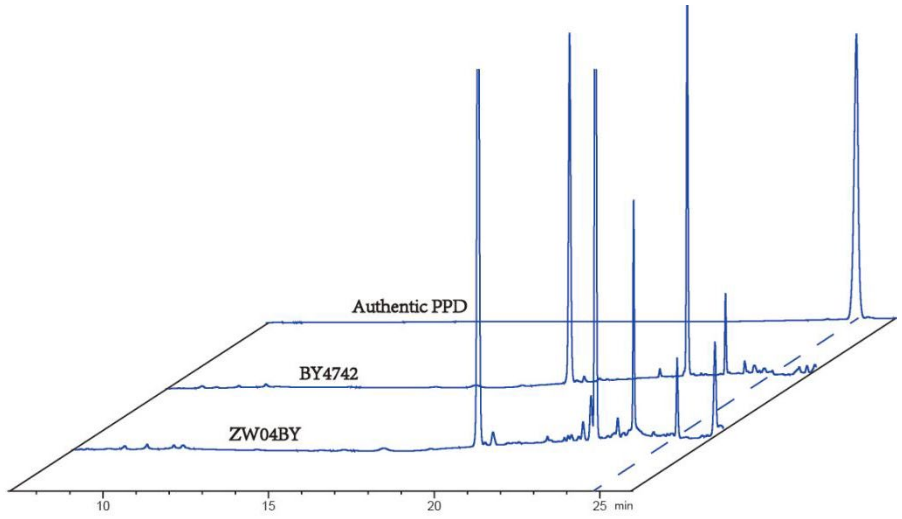

Supplement: Supplementary file 4 [file Data_Sheet_1.pdf]
