# Supplementary material for: Reconstruction of engineered yeast factory for high yield production of ginsenosides Rg3 and Rd
Source: Front Microbiol. 2023 Jun 19;14:1191102. doi: 10.3389/fmicb.2023.1191102 (PMC10315489; doi:10.3389/fmicb.2023.1191102)
Supplement: Supplementary file 5 [file Data_Sheet_2.docx]

**Supplementary Sequences**

>PnUGT31（Syn）

ATGGATAACCAGAAGGGTAGAATTTCCATTGCTTTGTTGCCATTTTTGGCCCATGGTCATATTTCTCCATTTTTCGAATTGGCTAAGCAGTTGGCTAAGAGAAACTGTAACGTTTTCTTGTGTTCTACGCCCATCAACTTGTCCTCTATTAAGAACAAAGATTCCTCCGCCTCTGTTAAGTTGGTTGAATTGCATTTGCCATCCTCTCCAGATTTGCCACCACATTATCATACAACTAATGGTTTGCCATCTCACTTGATGGTTCCATTGAGAAATGCTTTCGAAACTGTTGGTCCAACCTTCTCCGAAATTCTGAAAACTTTGAACCCAGACTTGCTGATCTACGATTTTAATCCATCTTGGGCTCCAGAAATTGCCTCCTCACATAATATTCCAGCTGTTTACTTTTTGACTACCGCTGCTGCTTCTTCCTCTATTGGTTTACATGCTTTTAAGAACCCAGGTGAGAAATACCCATTTCCAGATTTCTACGACAACTCCAACATTACTCCAGAACCACCATCTGCTGATAACATGAAGTTGTTGCATGATTTCATTGCCTGCTTCGAAAGATCCTGCGATATTATCTTGATTAAGTCCTTCAGAGAATTGGAGGGCAAGTACATTGATTTGTTGTCTACCTTGTCCGACAAGACTTTGGTTCCAGTTGGTCCATTGGTTCAAGATCCAATGGGTCATAATGAAGATCCAAAGACCGAACAAATCATCAACTGGTTGGATAAGAGAGCTGAATCTACCGTTGTTTTCGTTTGTTTCGGTTCCGAATACTTCTTGTCCAACGAAGAATTGGAAGAAGTTGCCATTGGTCTGGAAATCTCTATGGTTAATTTCATCTGGGCCGTCAGATTGATTGAAGGTGAAAAAAAAGGTGTCTTGCCAGAAGGTTTCGTTCAAAGAGTTGGAGATAGAGGTTTGGTTGTTGAAGGTTGGGCTCCTCAAGCTAGAATTTTGGGTCATTCTTCTACTGGTGGTTTCGTTTCTCATTGTGGTTGGTCATCTATTGCCGAATCTATGAAGTTTGGTGTTCCAGTTATTGCTATGGCCAGACATTTGGATCAACCATTGAATGCTAAATTGGCTGCCGAAGTTGGTGTTGGTATGGAAGTTGTTAGAGATGATAACGGCAAGTATAAGAGAGAAGGTATCGCCGAAGTTATCAGAAAGGTTGTTGTTGAAAAGTCCGGTGAAGTCATTAGAAGAAAGGCTAGAGAATTGTCCGAGAAGATGAAGGAAAAAGGCGAACAAGAAATTGATAGAGCCGTTGAAGAACTGGTTCAAATCTGCAAGAAAAAGAAGGATGCCCAGTAG

>PnUGT53

ATGAAGTCAGAATTGATATTCGTGCCCGCCCCGGCCATCGGACACCTCGTGGGAATGGTGGAGATGGCTAAACTCTTCATCAGTCGACATGAAAACCTCTCGGTCACCGTCCTCATCGCGAAATTCTACATGGATACGGGGGTAGACAACTACAATAAATCACTCTTAACAAAGCCTACCCCGCGTCTCACAATTGTAAATCTCCCGGAAAGCGACCCCCAAAACTATATGCTCAAACCACGCCACGCCATCTTTCCTAGCGTCATCGAGACTCAGAAGACACACGTGCGAGACATAATATTAGGCATGACTCAGTCCGAGTCGACTCGGGTCGTTGGTTTGCTGGCTGACCTTTTGTTCATCAACATTATGGACATTGCCAATGAGTTCAATGTTCCAACTTATGTATACTCCCCTGCCGGAGCAGGTCATCTTGGCCTCGCGTTCCATCTCCAGACACTCAACGACAAAAAGCAAGATGTGACCGAGTTCAGGCACTCGGACACTGAGTTATTGGTACCGAGTTTTGCAAACCCGGTTCCCGCCGAGGTCTTGCCGTCGATGTATGTGGATAAAGAAGGTGGGTATGATTATTTGTTTTCATTGTTCCGGAGGTGCAGAGAGTCAAAGGCAATTATTATTAACACGTTTGAGGAGCTGGAACCCTATGCGATCAATTCCCTCCGGATGGATAGTATGATCCCTCCGATCTACCCGGTGGGACCCATACTAAATCTCAACGGTGATGGCCAAAACTCCGATGAGGCTGCTGTGATCCTTGGTTGGTTAGACGATCAACCACCTTCATCTGTGGTGTTTTTGTGCTTTGGTAGCTATGGAACCTTTCAAGAAAACCAGGTGAAGGAGATTGCAATGGGTCTAGAGCGCAGTGGGCATCGCTTCTTGTGGTCCTTGCGTCCGTCTATCCCTAAAGGCGAGACAAAGCTTCAGCTTAAATACTCAAATTTGGAAGAAATTCTCCCAGTCGGATTCTTGGACAGGACATCATGCGTCGGAAAAGTTATTGGATGGGCCCCGCAAGTGGCGGTGCTCGGACACGAGGCAGTCGGAGGGTTCCTGTCTCATTGTGGTTGGAATTCGACATTAGAGAGTGTGTGGTGTGGCGTGCCCGTCGCAACATGGCCAATGTACGGCGAGCAACAACTCAATGCTTTTGAGATGGTTAAGGAGTTGGGTATTGCGGTGGAAATTGAGGTGGACTATAAGAATGAATATTTTAACATGACGAATGATTTTATTGTTAGGGCAGAAGAAATTGAGACGAAAATAAAGAAGTTGATGATGGATGAAAAGAATAGTGAAATAAGGAAGAAGGTAAAGGAAATGAAAGAAAAGAGTAGGCTTGCAATGTCTGAGAATGGATCATCTTATAATTCCTTGGCGAAACTATTTGAGGAAATTATGTGA

>Pn1-31

TCAGGAGGACACAAGCTTTGAAATGAACTCATCAATATTCTTATCAGAGCTACCCCCTTTCTCCATTGCCTCCACAGCCAACCCCTTCCATTTCAAAGCATTCCCTCTAAGCTCATCCCCTATTTCCCCCTCCATAACATCCTTTATACAGTTAGCAACTTCTCCCCTCAAAAGAACCCCATTTTCACCAATCGGAACCTGAACCCCAATTCGCCATACATCCACGATATACTTGGCATTAACAGGCTGATCAAACTGTTGTGGCACTGCCACCATTGGCACGCCCAAACTCAATGCCTCAACCGTCGAGTTCCAACCACAATGAGTCATAAAACTACCTATAGATTTATGTGACAAAACTTCGAGTTGAGGGCACCAACTCACTATTAGGCCTTTTTCCTCTGCCTCTGCCAAGAAATCGCTAGAGAGCTTTGTACGTTCGGATTCTATAACAACCCATATGAAGTGATATTTGCTGTGTAACAAGCCCCATGCTACTTCGTCCATGTAGTCATCGCCGAGGTTGTGCTTCACGCTCCCGAATGAGGCGTAGACTACCGAGCCAGGGTCTTTGGCGTCTAGCCACTTGATGCACGATCCGACGTCTGCCTTGTAGAAATTAATGTCATTGTCTTTGTCATTTGGTAATTGTTTGTCTAAGTACATTGAAGGGATTAATGGTCCGATGGATGTAATTGGCCATTGGCTAGATAGCCATTTTGCTTCCTCATTTTCAAGCTTATCAAAAGTATTGAAAAAGATCCAATCCGCTTTATCAAGATCTGAAAACTGATTCCCCAAATGCTCAAAGAGATCAGGAAAGGTGCCAGTCCGAATAATTGGCAAATCATTTCTCCCCAACAATGGAATTGAAGGCAATGAAACAGAAGTCAACCCTTCCTCAGGAGGTATCTTCAATTTCCCTAAGCGAGCATTATAATAAATGGCACCAACAGCACAAGAGTGAGTGAAAAATGGAGCTCCTTTTACCCCAAGTTGGTGGCACATTTCTACCCGAGATGGATATAATGAATCGACCACTAGCAAATTAACAGGGTAGCCATTTGTTTTTTGCTTCTCGAGTAGGTCACTCAGTTTATTTGTGACCTTGAGCTTTACAACTCCCATATAAGCCCGCATGCTGTATGGCATCTCATCATCTTTATCAAAATCAAAGGAGATGCACTCAGTGTTGATCAATGAGTTGTTTATTTGCATAGAATCTCGAATCTCAGCCGGAAGAACTATCGTGATTCGCACGCCTTTCCAGGCTAAACGCTTGACGAATTGCATCATTGGGCTCATGTGGCCTTGAGCTGGGAATGGGATGAACATAATGTGAGTTTTGCTCAACATTTCTCTGTCCAT

>SynUGT50

ATGGAAAGAGAAATGTTGTCTAAGACCCATATTATGTTCATTCCATTTCCAGCTCAAGGTCATATGTCTCCAATGATGCAATTCGTTAAAAGATTAGCTTGGAAGGGTGTTCGTATTACCATTGTTTTGCCAGCTGAAATTAGAGATTCTATGCAAATTAACAACTCATTAATTAACACTGAATGTATCTCTTTCGACTTCGATAAGGATGATGAAATGCCATACTCAATGAGAGCTTACATGGGTGTCGTCAAGTTAAAGGTTACTAATAAGTTGTCTGATTTGTTAGAAAAACAAAAGACTAATGGTTACCCAGTTAACTTGTTGGTTGTGGATTCTTTGTATCCATCCAGAGTCGAAATGTGTCATCAACTAGGTGTTAAAGGTGCTCCATTTTTCACTCACTCTTGTGCTGTTGGTGCTATCTACTATAACGCTAGATTGGGTAAATTGAAGATTCCACCAGAAGAAGGTTTAACTTCTGTTTCTTTGCCATCTATTCCATTGTTGGGTAGAAATGATTTGCCAATTATTAGAACAGGTACCTTTCCAGATTTGTTTGAACATTTGGGTAATCAATTCTCTGATTTGGATAAGGCTGACTGGATCTTTTTCAATACTTTCGATAAGCTAGAAAATGAAGAAGCTAAGTGGTTGTCCTCTCATTGGCCAATTACTTCCATTGGTCCATTGATCCCATCCATGTACTTAGATAAACAATTACCTAACGACAAGGATAACGATATTAACTTTTATAAGGCTGACGTCGGTTCTTGTATTAAGTGGTTGGATGCTAAGGATCCAGGTTCTGTCGTTTATGCATCTTTCGGTTCCGTTAAGCATAACCTTGGTGACGATTATATGGATGAAGTTGCCTGGGGTTTATTGCATTCTAAGTACCATTTCATCTGGGTCGTGATTGAATCTGAAAGAACTAAGTTATCTTCTGATTTCTTAGCTGAAGTTGAAGCTGAAGAAAAGGGTTTGATTGTCTCCTGGTGTCCACAATTAGAAGTTTTGTCACATAAGTCTATTGGTTCATTCATGACTCATTGTGGTTGGAACTCTACCGTCGAAGCTTTGTCTTTGGGTGTTCCAATGGTTGCAGTTCCTCAACAATTTGATCAACCTGCTAATGCTAAATACATTGTTGACGTTTGGAGAATCGGTGTTCAAGTCCCAATTGGTGAAAACGGTGTTCTGTTGAGAGGTGAAGTTGCTAACTGTATCAAAGATGTTATGGAAGGTGAAATTGGCGATGAATTGAGAGGTAACGCTTTGAAATGGAAAGGTTTGGCTGTTGAAGCTATGGAAAAGGGTGGTTCCAGTGATAAGAATATTGATGAATTCATCTCTAAATTGGTTTCTTCC

>PnUGT31

MDNQKGRISIALLPFLAHGHISPFFELAKQLAKRNCNVFLCSTPINLSSIKNKDSSASVKLVELHLPSSPDLPPHYHTTNGLPSHLMVPLRNAFETVGPTFSEILKTLNPDLLIYDFNPSWAPEIASSHNIPAVYFLTTAAASSSIGLHAFKNPGEKYPFPDFYDNSNITPEPPSADNMKLLHDFIACFERSCDIILIKSFRELEGKYIDLLSTLSDKTLVPVGPLVQDPMGHNEDPKTEQIINWLDKRAESTVVFVCFGSEYFLSNEELEEVAIGLEISMVNFIWAVRLIEGEKKGVLPEGFVQRVGDRGLVVEGWAPQARILGHSSTGGFVSHCGWSSIAESMKFGVPVIAMARHLDQPLNAKLAAEVGVGMEVVRDDNGKYKREGIAEVIRKVVVEKSGEVIRRKARELSEKMKEKGEQEIDRAVEELVQICKKKKDAQ

>PnUGT53

MKSELIFVPAPAIGHLVGMVEMAKLFISRHENLSVTVLIAKFYMDTGVDNYNKSLLTKPTPRLTIVNLPESDPQNYMLKPRHAIFPSVIETQKTHVRDIILGMTQSESTRVVGLLADLLFINIMDIANEFNVPTYVYSPAGAGHLGLAFHLQTLNDKKQDVTEFRHSDTELLVPSFANPVPAEVLPSMYVDKEGGYDYLFSLFRRCRESKAIIINTFEELEPYAINSLRMDSMIPPIYPVGPILNLNGDGQNSDEAAVILGWLDDQPPSSVVFLCFGSYGTFQENQVKEIAMGLERSGHRFLWSLRPSIPKGETKLQLKYSNLEEILPVGFLDRTSCVGKVIGWAPQVAVLGHEAVGGFLSHCGWNSTLESVWCGVPVATWPMYGEQQLNAFEMVKELGIAVEIEVDYKNEYFNMTNDFIVRAEEIETKIKKLMMDEKNSEIRKKVKEMKEKSRLAMSENGSSYNSLAKL

>Pn1-31

MDREMLSKTHIMFIPFPAQGHMSPMMQFVKRLAWKGVRITIVLPAEIRDSMQINNSLINTECISFDFDKDDEMPYSMRAYMGVVKLKVTNKLSDLLEKQKTNGYPVNLLVVDSLYPSRVEMCHQLGVKGAPFFTHSCAVGAIYYNARLGKLKIPPEEGLTSVSLPSIPLLGRNDLPIIRTGTFPDLFEHLGNQFSDLDKADWIFFNTFDKLENEEAKWLSSQWPITSIGPLIPSMYLDKQLPNDKDNDINFYKADVGSCIKWLDAKDPGSVVYASFGSVKHNLGDDYMDEVAWGLLHSKYHFIWVVIESERTKLSSDFLAEAEEKGLIVSWCPQLEVLSHKSIGSFMTHCGWNSTVEALSLGVPMVAVPQQFDQPVNAKYIVDVWRIGVQVPIGENGVLLRGEVANCIKDVMEGEIGDELRGNALKWKGLAVEAMEKGGSSDKNIDEFISKLVSS.

>SynUGT50

MEREMLSKTHIMFIPFPAQGHMSPMMQFVKRLAWKGVRITIVLPAEIRDSMQINNSLINTECISFDFDKDDEMPYSMRAYMGVVKLKVTNKLSDLLEKQKTNGYPVNLLVVDSLYPSRVEMCHQLGVKGAPFFTHSCAVGAIYYNARLGKLKIPPEEGLTSVSLPSIPLLGRNDLPIIRTGTFPDLFEHLGNQFSDLDKADWIFFNTFDKLENEEAKWLSSHWPITSIGPLIPSMYLDKQLPNDKDNDINFYKADVGSCIKWLDAKDPGSVVYASFGSVKHNLGDDYMDEVAWGLLHSKYHFIWVVIESERTKLSSDFLAEVEAEEKGLIVSWCPQLEVLSHKSIGSFMTHCGWNSTVEALSLGVPMVAVPQQFDQPANAKYIVDVWRIGVQVPIGENGVLLRGEVANCIKDVMEGEIGDELRGNALKWKGLAVEAMEKGGSSDKNIDEFISKLVSS
